# Quantitative cardiac CT perfusion: physiologically-inspired model and identifying microvascular disease from discordant CTA CAD-RADS

**DOI:** 10.3389/fcvm.2025.1621443

**Published:** 2025-11-10

**Authors:** Hao Wu, Yingnan Song, Ammar Hoori, Juhwan Lee, Sadeer Al-Kindi, Wei-Ming Huang, Chun-Ho Yun, Chung-Lieh Hung, Sanjay Rajagopalan, David L. Wilson

**Affiliations:** 1 Department of Biomedical Engineering, Case Western Reserve University, Cleveland, OH, United States; 2 Harrington Heart and Vascular Institute, University Hospitals Cleveland Medical Center, Cleveland, OH, United States; 3 Department of Radiology, MacKay Memorial Hospital, Taipei, Taiwan; 4 Division of Cardiology, Department of Internal Medicine, MacKay Memorial Hospital, Taipei, Taiwan; 5 School of Medicine, Case Western Reserve University, Cleveland, OH, United States; 6 Department of Radiology, Case Western Reserve University, Cleveland, OH, United States

**Keywords:** cardiac CT perfusion, deep learning, image processing, coronary CT angiography, flow-limiting stenosis, microvascular disease

## Abstract

**Objective:**

Use our advanced, physiologically inspired cardiac CT perfusion (CCTP) software to distinguish ischemia due to obstructive disease vs. microvascular disease (MVD).

**Background:**

Previously validated advanced CCTP methods were used. We interpreted results to identify flow-limiting stenosis [i.e., obstructive-lesion & low myocardial blood flow (MBF)] vs. microvascular disease (i.e., no-obstructive-lesion & low-MBF).

**Methods:**

We retrospectively evaluated 104 patients with suspected CAD, including 18 with diabetes, who underwent CCTA + CCTP. Whole heart and territorial MBF was assessed using our automated pipeline for CCTP analysis that included beam hardening correction; temporal scan registration; automated segmentation; fast, accurate, robust MBF estimation; and visualization. Stenosis severity was scored using the CCTA coronary-artery-disease-reporting-and-data-system (CAD-RADS), with obstructive stenosis deemed as CAD-RADS ≥ 3.

**Results:**

We established a threshold MBF (MBF = 200-mL/min-100 g) for normal perfusion. In patients with CAD-RADS ≥ 3 (obstructive disease), 28/37(76%) patients showed ischemia in the corresponding territory. On a per-vessel basis (*n* = 256), MBF showed a significant difference between territories with and without obstructive stenosis (165 ± 61 mL/min−100 g vs. 274 ± 62 mL/min−100 g, *p* < 0.05). A significant negative rank correlation (*ρ* = −0.53, *p* < 0.05) between territory MBF and CAD-RADS was seen. Two patients with obstructive disease had normal perfusion, suggesting collaterals and/or hemodynamically insignificant stenosis. Among diabetics, 10 of 18 (56%) demonstrated diffuse ischemia consistent with MVD. Among non-diabetics, only 6% had MVD. Sex-specific prevalence of MVD was 21%/24% (M/F).

**Conclusion:**

CCTA in conjunction with a new automated quantitative CCTP approach can determine the distinction of ischemia due to obstructive lesions vs. MVD.

## 1 Introduction

Current imaging options for coronary artery disease are expensive, incomplete, and may often require multiple modalities to confirm functionally significant coronary artery disease (CAD). Invasive coronary angiography (ICA) is the clinical standard for detecting anatomical obstructive CAD, but its correlation with myocardial ischemia is poor, leading to the use of pressure wire functional flow reserve (FFR) (1). The 2024 chest pain guidelines suggest documenting anatomy with a coronary CT angiography (CCTA) exam when less obstructive CAD is suspected. If obstructive CAD is suspected, confirmation of ischemia with a non-invasive function test (e.g., stress echocardiography, SPECT, PET, or cardiac MRI) for additional validation is recommended (2). Many ICAs are negative for obstructive CAD, with over 60% of elective ICAs showing no hemodynamically significant obstructive disease (3). With ≈1M ICAs in the US every year, an improved, non-invasive “gatekeeper strategy” could reduce many unnecessary ICAs. Many patients have been noted to have diffuse coronary microvascular disease or dysfunction (MVD) resulting in reduced flow in the absence of a functionally relevant stenosis (4–6). Noted risk factors include diabetes, hypertension, and post-menopause. A recent systematic review noted a significant percentage (41%) of MVD in patients with non-obstructive CAD, necessitating careful differentiation as this condition does not warrant an interventional approach (6). An ideal gatekeeper evaluation should enable accurate and simultaneous delineation and distinction of functionally significant stenosis from MVD.

CCTA is a highly sensitive tool for detecting and excluding coronary artery stenosis. The use of CT-derived FFR methods (e.g., FFR_CT_ from HeartFlow) has improved discrimination of hemodynamically significant CAD (7, 8). Studies combining CCTP with CCTA show benefits as compared to CCTA alone (9, 10). The additional advantage of a combined CCTP + CCTA approach is that this combination can be used to identify microvascular disease (11) when there is ischemia but no obstructive disease. Increased acceptability of stress CCTP will require key technical elements: appropriate scanner availability, methods to correct beam hardening artifacts, low-dose imaging, automated and pragmatic tools for scan registration and segmentation, and accurate MBF estimation.

In this report, we refined and applied a highly automated pipeline with multiple innovations developed by our group to quantitatively analyze CCTP images (12–16). To correct for beam hardening artifacts, we used a previously described image-based automated beam hardening correction (ABHC) (12). We used a robust, physiology-based perfusion model (RPM1), which proved to be more accurate than eight other approaches with realistic simulated data (13). To reduce the effects of noise and obtain better MBF estimates, we developed the simple linear iterative clustering algorithm with robust perfusion quantification (SLICR) method (14). Here, we used these methods to analyze patients with both dynamic CCTP and CCTA images, where the latter were evaluated using the Coronary Artery Disease-Reporting and Data System (CAD-RADS) (17). CCTP assessments were evaluated with consideration to the presence or absence of obstructive disease as determined from CAD-RADS on a coronary territory basis. Effects of gender and diabetes were analyzed.

## 2 Materials and methods

### 2.1 Dataset

This study was approved as a retrospective study of de-identified data by the institutional review board of Mackay Memorial Hospital, Taiwan (19MMHIS275e 08/01/2019). Images were acquired starting in 2013 at Mackay Memorial Hospital, Taipei, Taiwan, as part of clinical protocol and shared under a data use agreement. The population consisted of 148 patients with suspected CAD who underwent CCTA and stress CCTP. We excluded 44 patients based on the following criteria: (1) age <20 years, (2) coronary artery bypass grafting, (3) acute or old myocardial infarction, (4) complete left bundle branch block, and (5) inadequate datasets such as poor image quality of CCTA or insufficient CCTP analysis. Sequential CCTA and stress dynamic CCTP were performed.

All patients underwent a dual-source CT system (Somatom Definition Flash; Siemens Healthineer, Forchheim, Germany) with a 128-slice detector. CCTA and CCTP acquisitions are described as follows: A non-contrast scout image was followed by a timing bolus acquisition at the aortic root using 15-mL of contrast (Iopamidol 370; Bracco) at 5 mL/sec, plus 20-mL saline via a dual-syringe injector. The CCTA acquisition started 8-seconds after peak contrast enhancement in the ascending aorta with 50-mL of contrast medium, followed by 40-mL of saline, injected at 5.0-mL/sec. A stress CCTP was performed 10-minutes after completing the CCTA scan. The dual-source scan was performed for 30-seconds, starting 6-seconds after the power injector started to administer the contrast medium. Dipyridamole (0.56-mg/kg) was administered over 4-minutes, with the stress dynamic CCTP conducted 6-minutes after starting the dipyridamole infusion. A total of 50-mL of contrast medium was injected, followed by 60-mL of saline, both at a rate of 5.0-mL/sec, using the same power injector. Post-stress CCTP, aminophylline (3-mg/kg) was administered intravenously over 2-minutes. Details of the CT acquisition/reconstruction settings are provided in the supplementary material.

### 2.2 CCTA image analysis

The CCTA images were evaluated by two experienced readers (CHY, WMH, with 16 and 6 years of cardiac CT experience, respectively). Readers were blinded to the subject's clinical presentation and history. Any disagreement was solved by consensus. On a per vessel basis, stenoses were classified as the expert consensus document of the Society of Cardiovascular Computed Tomography: CAD-RADS = 0 (0% luminal diameter stenosis), CAD-RADS = 1 (1%–24% stenosis), CAD-RADS = 2 (25%–49% stenosis), CAD-RADS = 3 (50%–69% stenosis), CAD-RADS = 4 (70%–99% stenosis), and CAD-RADS = 5 (100% stenosis). CAD-RADS ≥ 3 was considered as obstructive stenosis (17).

### 2.3 Quantitative CT perfusion pipeline

**Figure 1:**
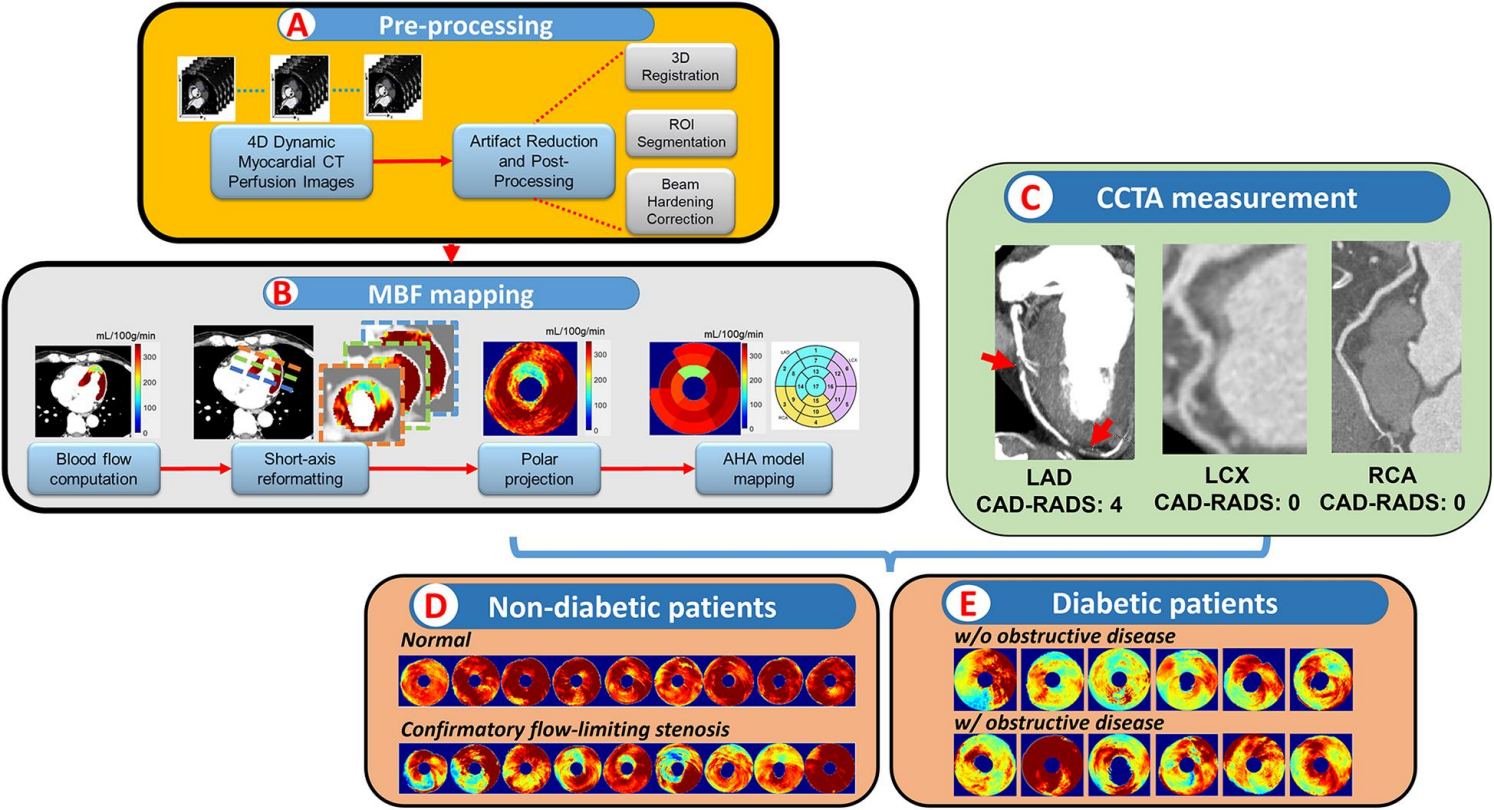
Pipeline of combinatory CCTP&CCTA analysis. In **(A, B)**, a highly automatic prototype software analyzed the CCTP data and produced a polar map then mapped to AHA segments. Vessel-wised CAD-RADS were measured in CCTA **(C)**. Patients were then separated into different subgroups and analyzed based on different conditions **(D, E)**.

We refined a highly automated prototype software to quantitatively analyze CCTP data based on our previous works (12–16). The processing pipeline is shown in Figures 1A,B. The quantitative CT perfusion pipeline includes: (i) aorta region of interest (ROI) detection from unregistered data, (ii) temporal scan registration, (iii) myocardium and aorta segmentation, (iv) automatic beam hardening correction, (v) MBF computation on axial images, (vi) MBF polar map conversion, and (vii) generating American Heart Association (AHA) segment report (18). Myocardial blood flow (MBF) was estimated using the Robust Physiological Model (RPM1), a simplified Johnson-Wilson model with three free parameters (time delay, MBF, and decay constant), which we have previously validated against other approaches (13). We excluded a segment from the AHA model if the number of pixels on the polar map was less than 30% of the segment area. To determine the representative absolute MBF for each coronary territory (LAD, RCA, and LCX), we evaluated all pairs of spatially adjacent AHA segments within the territory and selected the pair with the lowest average MBF to represent the characteristic MBF of that territory. For instance, in the LAD territory, the adjacent segment combinations were [1, 2], [1, 7], [1, 8], [2, 7] [2, 8], [7, 8], [13, 14], [7, 13], [8, 13], and [8, 14]. The pair with the lowest mean MBF was used as the representative LAD MBF. The relative MBF for each AHA segment was then calculated by normalizing to the coronary territory with the highest representative average MBF. This approach reduces the effect of local noise and improves robustness compared to using a single segment. Details are in the supplemental document.

We trained two convolutional neural networks, one for myocardium and aorta segmentation, and one for landmarks (interventricular septum and center of left ventricle) detection in short-axis data for MBF polar map conversion, respectively. Two analysts manually annotated the CCTP images. The myocardium and aorta were segmented on peak enhancement volume in axial images. The landmarks for polar map conversion were labeled in short-axis volume with peak enhancement. Analyst1 labeled the entire dataset, including 104 patients, to train the two convolutional neural networks. Analyst2 labeled 15 patients to perform an inter-observer study and evaluate the performance of the pipeline. The details of each step are in supplementary material.

### 2.4 Statistical analysis

We used the MATLAB statistic toolbox for statistical analysis (19). Continuous variables were expressed as means ± standard deviations while categorical variables were expressed as frequency and ratio (%). The unpaired Student's *t*-test was used for normally distributed variables to compare the means between the two groups. The Spearman's rank correlation coefficient (*ρ*) evaluated correlations between continuous MBF and ordinal CAD-RADS. To identify optimal stress MBF cutoffs on a per-territory basis, we restricted the ROC analysis to unequivocal cases (CAD-RADS = 0 or 4) to minimize misclassification. CAD-RADS = 3 cases were excluded because intermediate stenosis may not always correspond to abnormal flow. A bootstrap-corrected Receiver Operating Characteristic (ROC) curve analysis with 1,000 bootstrapped samples was performed, and the mean Youden index was used to determine optimal stress MBF cutoffs on a per-territory basis (20, 21). The optimal MBF threshold was 200 mL/min−100 g. Differences were statistically significant at *P* < 0.05.

## 3 Results

### 3.1 Software validation

We quantitatively evaluated each intermediate and final output of our software. Segmentation and landmark identification for AHA segments were acceptable as now described. The automated segmentation module for the myocardium and aorta had average Dice scores of 0.90 ± 0.04 and 0.92 ± 0.02, respectively. Mis-segmentation mainly occurred around the apex in patients with thinner myocardium. In our modified AHA-16 model, the apical cap was excluded from MBF analysis. Euclidean distances were 2.3 ± 1.9 mm, 2.9 ± 4.1 mm, and 3.8 ± 2.7 mm for the center of LV, upper septum, and bottom septum, respectively. We compared results of automatic and manual segmentation on territory MBF (Supplementary Figures S4, S5). Agreement between analysts was excellent (R = 0.99, *p* < 0.001), with the automatic method showing similar agreement (R = 0.98, *p* < 0.001 for both analysts). Details are in the supplemental document.

### 3.2 Clinical image data analysis

**Figure 2:**
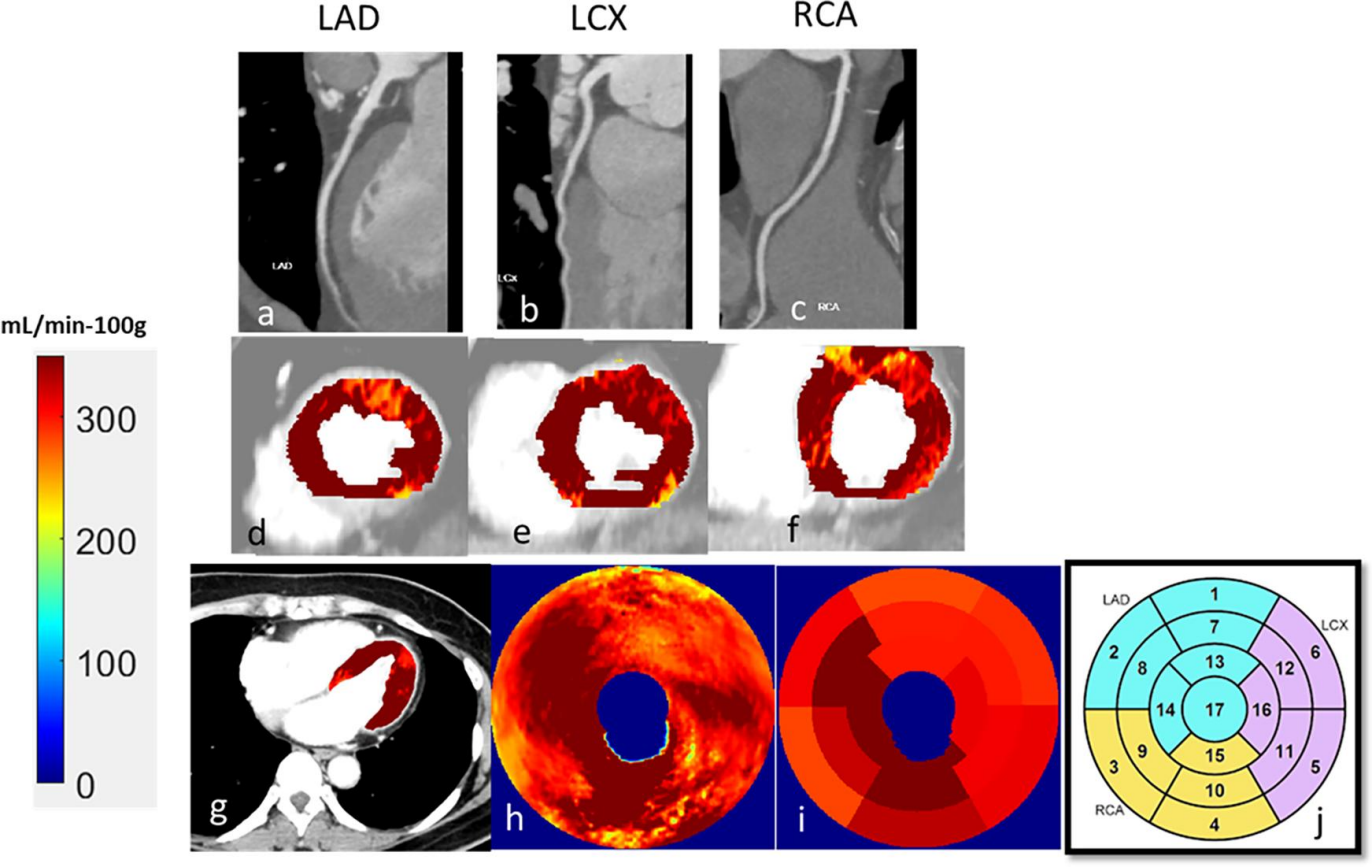
CCTA and CCTP analysis of a 47-year-old male patient with typical angina chest pain. CCTA **(a–c)** showed no luminal stenosis for all arteries. CCTP showed a normal stress MBF for all territories **(d–i)**. The AHA sectors in **(i)** had a stress MBF of 320 ± 30 mL/min−100 g. The map between AHA segments and the corresponding coronary artery is shown in **(j)**.

**Figure 3:**
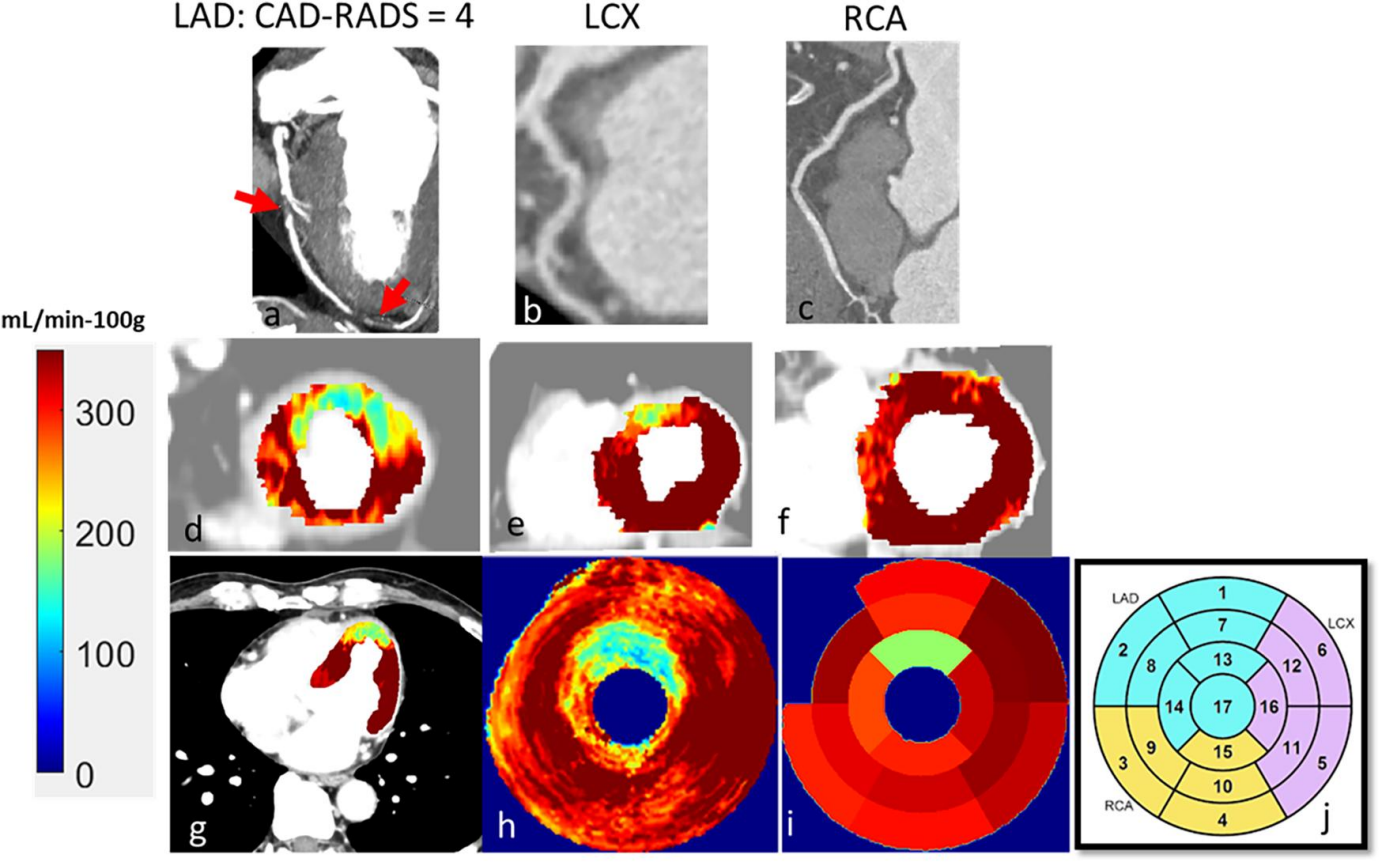
A 60-year-old male patient with typical angina chest pain. CCTA **(a–c)** showed >70% luminal narrowing of the LAD (1-vessel disease). CCTP showed a perfusion defect with an abnormal stress MBF of 178 mL/min−100 g in the territory supplied by the LAD **(d–i)**. The map between AHA segments and the corresponding coronary artery is shown in **(j)**.

The characteristics of the 104 patients are presented in Table 1. Patient age was 58 ± 12 years, and 41 (39%) were female. Of the 104 patients, 22 (17%) showed CAR-RADS ≥ 3 in one vessel, 13 (12%) showed CAR-RADS ≥ 3 in two vessels, and 2 (2%) showed CAR-RADS ≥ 3 in all three vessels. Example image analyses are shown in Figures 2, 3. In a patient with CAD-RADS = 0 (Figure 2), MBF was uniformly high on all views from apical to basal slices (d, e, and f) and on the polar map and the AHA-16 displays (h and i, respectively). In Figure 3, we show a patient with CAD-RADS = 4 with high-grade stenosis in the LAD and D1. The patient has concordant reduced MBF in the LAD territory.

**Table 1:** Baseline characteristics of the patients.

Parameters	All patients (*n* = 104)
Demographic parameters	
Age—means ± SD	58 ± 12
Female—no./total no. (%)	41/104 (39)
Cardiovascular risk factors—no./total no. (%)	
Angina	82/104 (78)
Hypertension	55/104 (52)
Dyslipidemia	58/104 (55)
Current or former smoking	16/104 (15)
Diabetes	18/104 (17)
CCTA findings—no./total no. (%)	
CAD-RADS < 3	67/104 (64)
1 vessel CAD-RADS ≥ 3	22/104 (21)
2 vessel CAD-RADS ≥ 3	13/104 (12)
3 vessel CAD-RADS ≥ 3	2/104 (2)

### 3.3 MBF cutoff

The optimal stress MBF threshold for abnormal flow was 200 mL/min−100 g (95% CI: 179.2–220.8 mL/min−100 g). Differences were statistically significant at *P* < 0.05. (Supplementary Figure S6). To address potential clustering of coronary territories within the same patient, we additionally performed patient-level stratified bootstrap analysis. Results were consistent, with the optimal threshold remaining 200 mL/min−100 g (95% CI: 174.3–221.2 mL/min−100 g).

### 3.4 Perfusion patterns as a function of CAD-RADS and presence of diabetes

**Figure 4:**
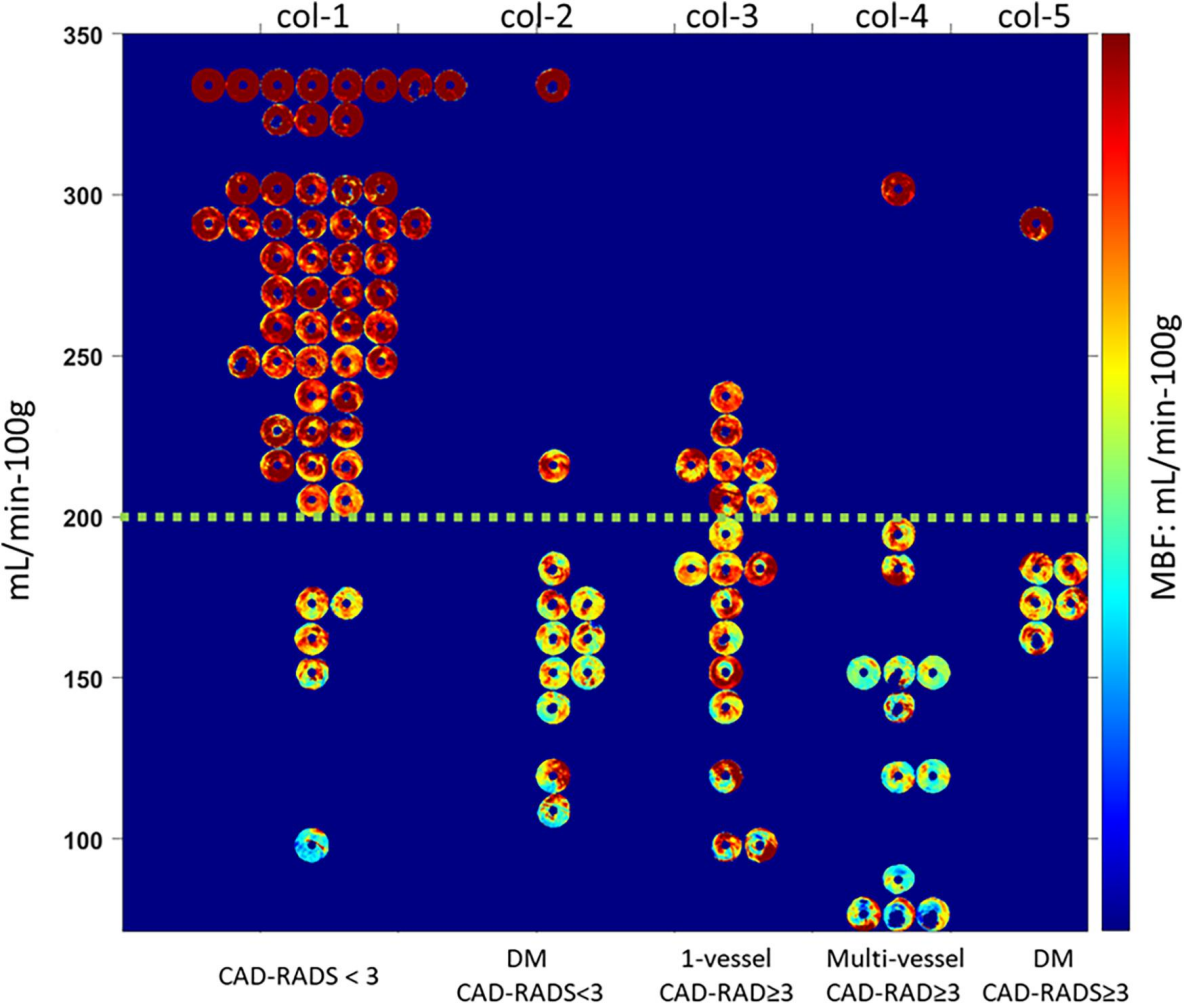
Polar maps of myocardial blood flow (MBF) for 104 patients, divided into five groups based on diabetes status and the number of arteries with obstructive stenosis. **(a)** No diabetes, no obstructive stenosis (CAD-RADS < 3). **(b)** Diabetes, no obstructive stenosis (DM-CAD-RADS < 3). **(c)** No diabetes, obstructive stenosis in one vessel (1-vessel-CAD-RADS ≥ 3). **(d)** No diabetes, obstructive stenosis in multiple vessels (multi-vessel-CAD-RADS ≥ 3). **(e)** Diabetes, obstructive stenosis (DM-CAD-RADS ≥ 3). The green dashed line indicates the abnormal MBF threshold (200 mL/min−100 g). Polar maps are consistent with patient groupings. (See text for details.).

When polar maps of all 104 patients were categorized into sub-groups, several observations were evident (Figure 4). Focusing on patients without diabetes mellitus (DM) in columns 1, 3, and 4), who have a lower prevalence of MVD, stenosis severity agreed well with MBF. In column 1, for such patients with CAD-RADS < 3, 50 out of 55 (90%) showed normal global MBF. Four of the remaining 5 showed reduced flow in one territory despite having CAD-RADS of 1 or 2. One patient demonstrated global, severely low MBF, suggestive of diffuse MVD. For those with CAD-RADS ≥ 3 in one vessel, 11 out of 18 (61%) patients showed an abnormally low MBF territory that corresponded to the obstructed vessel. For CAD-RADS ≥ 3 in multiple vessels, 12 out of 13 (93%) patients had at least one territory with abnormal MBF corresponding to an obstructed vessel. Notably, global abnormal MBF was more common in the multi-vessel group than in the 1-vessel group (10 vs. 1, respectively), with lower mean global MBF in the multi-vessel group (158 ± 47 mL/min−100 g) as compared to the 1-vessel group (209 ± 68 mL/min−100 g, *p* < 0.05).

Continuing with the analysis of patients without diabetes, there are some interesting discordances between CAD-RADS and MBF. For CAD-RADS ≥ 3 (columns 3 and 4), we found that 8 out of 31 (26%) patients showed normal global MBF. In particular, there were 4 cases with CAD-RADS = 4 with normal MBFs. Surprisingly, one case of multivessel CAD-RADS ≥ 3 (column 4) showed very high perfusion without a deficit in the stress test. These discordant cases with preserved perfusion despite obstructive CCTA stenosis suggest that some lesions may be hemodynamically insignificant, potentially due to collateral supply or overestimation of severity by CCTA. Functional perfusion imaging provides important complementary information in such cases.

Patients with diabetes mellitus (columns 2 and 5) are analyzed separately, given the frequent association of MVD with diabetes. Among diabetic patients without obstructive disease (CAD-RADS < 3, column 2), 10 of 12 had low MBF, 1 had marginal MBF, and one had high MBF. The difference between column 2 (with DM) and column 1 (no DM) is extremely striking. In column 2, the low MBF (blue to blue-green color) often appears to surround the polar map, suggesting a global effect. These observations are highly suggestive of diffuse MVD. In patients with obstructive disease (CAD-RADS ≥ 3, column 5), 5 of 6 patients had low MBF, and 1 had high MBF. We should point out that the duration of diabetes diagnosis was unknown.

Given that being female is thought to be a risk factor for MVD, we analyzed gender as a variable. We identified 15 patients with low MBF and CAD-RADS < 3 as having MVD, regardless of diabetes. Among our study participants, 21% of males and 24% of females had MVD. A larger sample size is needed for a more thorough analysis.

For this cohort, for non-diabetic patients, MVD can nearly be excluded. That is, of the 55 non-diabetic patients with CAD-RADS < 3, only 5 had low MBF, and only one had a severely low MBF (<100 mL/min−100 g).

### 3.5 Territory analyses

**Figure 5:**
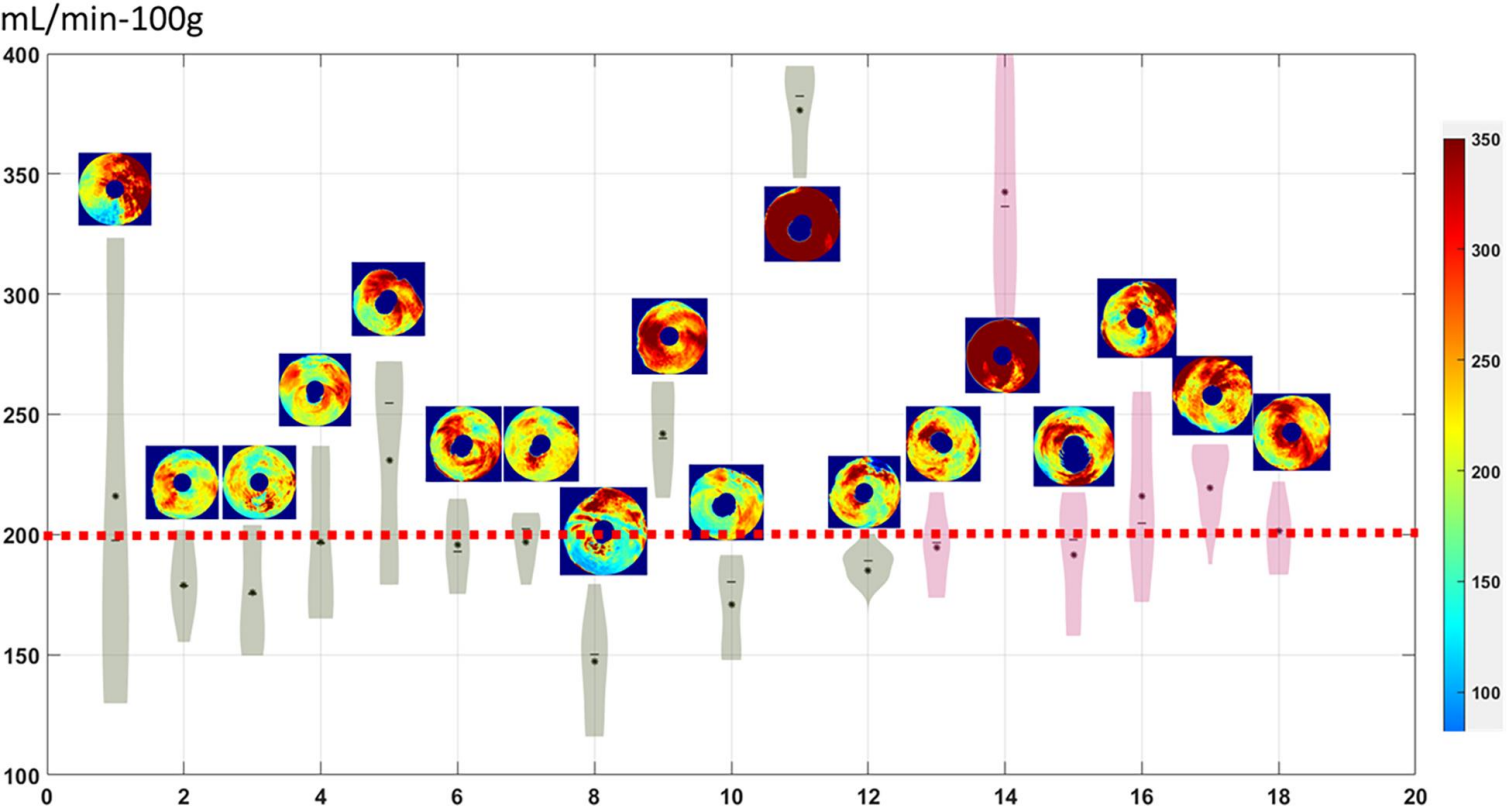
Polar map distribution of diabetic patients. Diabetic patients without and with obstructive disease are in green and purple, respectively. Ischemia is indicated when MBF distribution falls below the red dashed line. Patients 9, 11, and 14 showed normal MBF, while patients 1, 5, and 17 had local ischemia. The remaining 12 patients exhibited diffuse ischemia. Polar maps allow easy visual interpretation.

To further analyze the spatial distribution of MBF in diabetic patients on a per-territory basis (Figure 5). MBF distribution in each of the polar maps was evaluated for ischemia when below the red dashed line threshold. In total, 12 out of 18 (67%) diabetic patients were deemed to have diffuse ischemia, corresponding to a low, tight distribution of MBFs. Among those without obstructive disease (green, *n* = 12), 8 showed diffuse ischemia in over half of the segments, with global mean MBF below the optimal cutoff. Two showed ischemia in one territory, and two showed no ischemia. For diabetic patients with obstructive stenosis (purple, *n* = 6), four showed diffuse ischemia in multiple territories, one showed ischemia in one territory, and one showed no ischemia. Altogether, the patients with diabetes tended to have low MBF regardless of the presence of obstructive disease.

**Figure 6:**
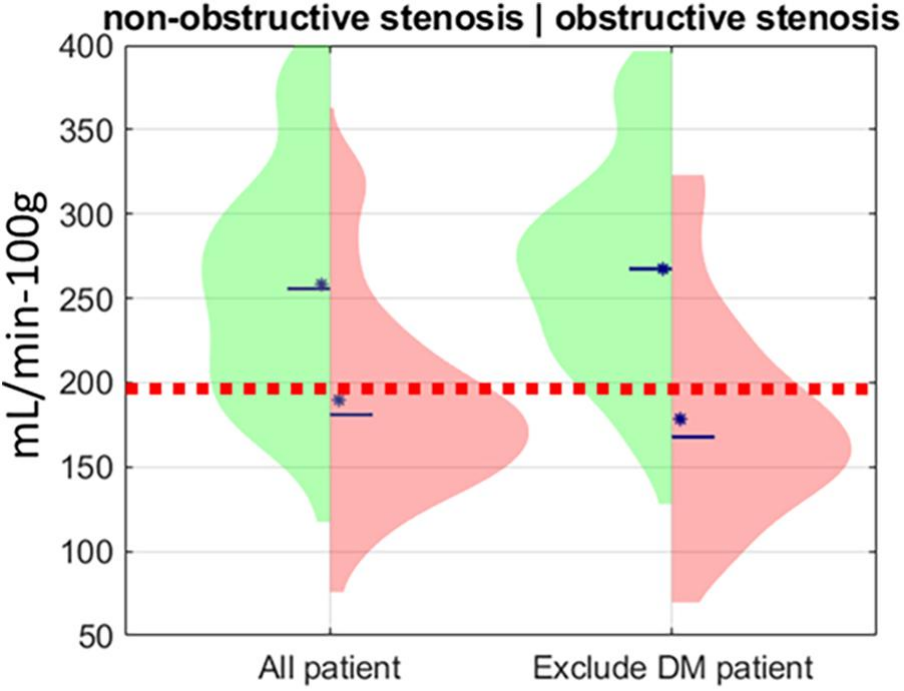
MBF distribution of individual territories without obstructive stenosis and with obstructive stenosis before and after excluding territories from diabetic patients. The red dashed line is the optimal MBF cutoff (200 mL/min−100 g). The blue asterisk is the median value of each distribution. The blue solid line is the mean value of each distribution.

To learn the relationship between MBF and stenosis severity in patients without diabetes, we continued with the territory analysis (Figure 6). Before excluding diabetic patients, mean MBFs were 259 ± 65 mL/min−100 g and 184 ± 58 mL/min−100 g in non-obstructed and obstructed territories, respectively, *p* < 0.001. After excluding diabetic patients, mean MBFs were 274 ± 62 mL/min−100 g and 165 ± 61 mL/min−100 g, respectively, *p* < 0.001. By excluding diabetic patients, we reduced the confounding effect of MVD on territory MBF and increased the difference between non-obstructed and obstructed territories from 75 to 109 mL/min−100 g, creating a clearer demarcation between territories with and without obstructive disease.

**Figure 7:**
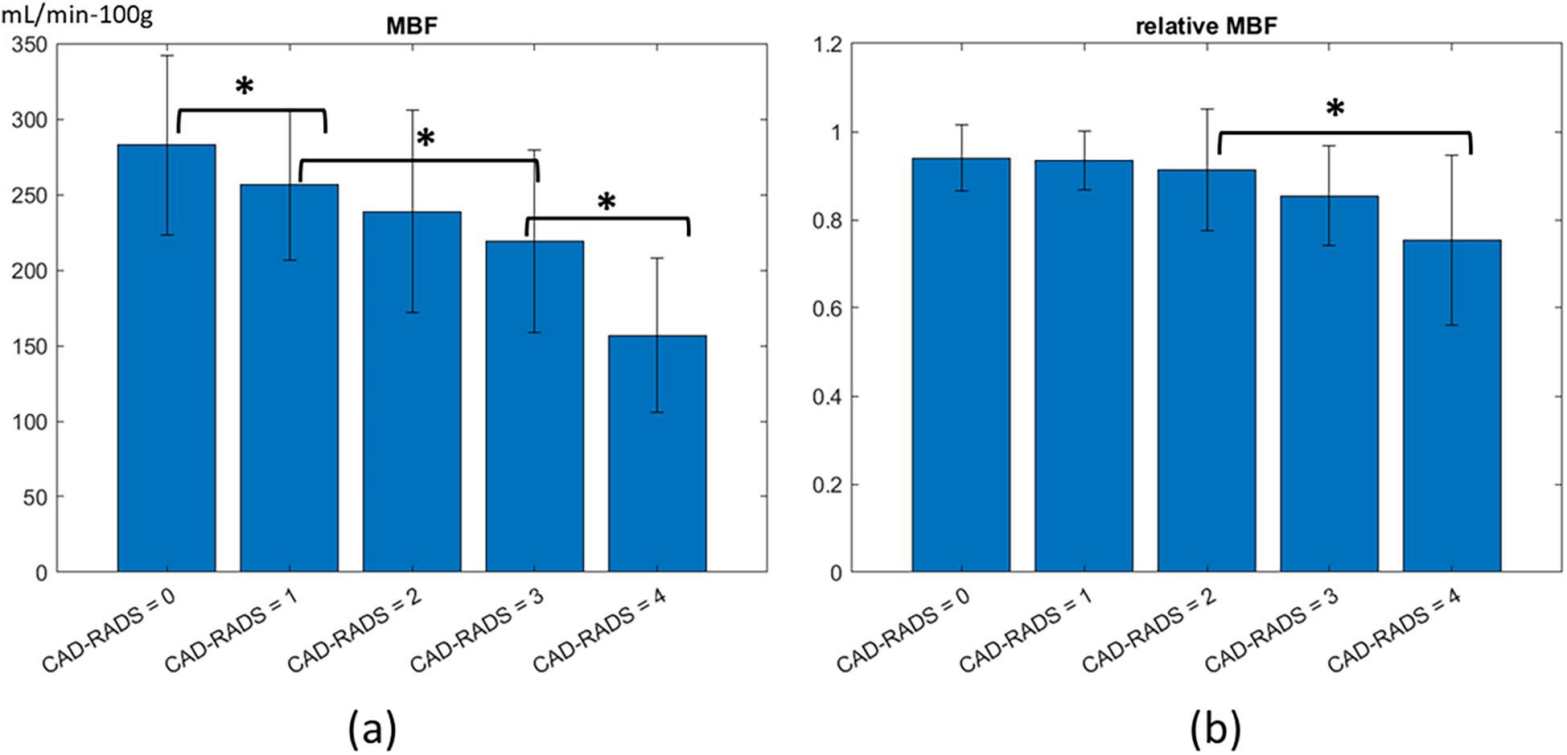
Association of stress MBF and CAD-RADS, analyzed per territories. **(a)** There is a moderate Spearman's rank correlation between absolute MBF and CAD-RADS score (*ρ* = −0.53, *p* < 0.05). **(b)** There is a weaker Spearman's rank correlation between relative MBF and CAD-RADS (*ρ* = −0.43, *p* < 0.05). The relative MBF was calculated by normalizing each AHA segment with the territory with the highest average MBF. Between groups, *t*-tests are performed with an asterisk indicating *P* < 0.05.

The agreement of territory-specific MBF and CAD-RADS stenosis severity was investigated with diabetic patients excluded to reduce any effect of MVD (Figure 7). We found a significant and negative correlation between the CAD-RADS of a vessel and the absolute MBF of the corresponding territory. MBF was highest in CAD-RADS = 0 (286 ± 59 mL/min−100 g) and lowest in CAD-RADS = 4 (156 ± 51 mL/min−100 g), with statistical significance (*p* < 0.05). When Spearman rank correlation was computed, there was a stronger correlation using absolute MBF than with relative MBF. A student *t*-test between groups of relative MBF measurements showed insignificant differences between most groups. When we included patients having diabetes, there was even a poorer correlation (*ρ* = −0.38, *p* < 0.05) between relative MBF and CAD-RADS (not shown), suggesting that the presence of diffuse MVD can confound the usage of relative MBF.

Further analyses of territories are done in the Supplemental document. The percentage of ischemic territories (MBF < 200 mL/min−100 g, blue color) increased with CAD-RADS, particularly for vessels with CAD-RADS = 3 and 4, where 49% and 13% did not show downstream ischemia, respectively (Supplementary Figure S7). For CAD-RADS = 1 and 2, there are still 21%, and 20% showed downstream ischemia, suggesting a microvascular disease. Finally, we analyzed the percentage of territories as a function of different MBF levels (Supplementary Figure S7), again excluding diabetic patients to limit the confounding effect of MVD on MBF. As described in the figure legend, there was good consistency between CAD-RADS scores and territory MBF.

## 4 Discussion

Our study demonstrates a new methodology for distinguishing flow-limiting stenosis from microvascular disease (MVD). We refined an automated pipeline for quantitative MBF in CCTP, incorporating beam hardening correction, temporal scan registration, segmentation, and a novel method for MBF assessment (12–16). Our enhanced automated pipeline facilitated precise MBF analysis using CCTP images, incorporating accurate myocardium and aorta segmentation for beam hardening correction and MBF computation, and automatic landmark localization for polar map generation from short-axis views. The automated results closely matched manual results (Supplementary Figures S4, S5).

We now review our MBF cut-off for ischemia (200 mL/min−100 g) to values reported in the literature for stress MBF for different modalities. PET scans, considered highly accurate, use values from 185 to 250 mL/min−100 g for obstructive stenosis (22, 23). Cardiovascular magnetic resonance (CMR) studies have cutoffs from 129 to 194 mL/min−100 g (24, 25). In our study, the optimal cutoff for CAD-RADS = 4 was 200 mL/min−100 g. While >50% stenosis is typically considered hemodynamically significant, we chose CAD-RADS = 4 to ensure unequivocal cases when deriving the MBF cutoff. The SPECIFIC study found an optimal MBF threshold of 142 mL/min−100 g for detecting obstructive CAD with CCTP (26). Our previous work showed this method significantly underestimates MBF in both simulated and *in vivo* studies (13), unlike our preferred method. Similarly, low MRI values may depend on the computational method used.

Our results show that in stress CCTP, the absolute MBF is better correlated to CAD-RADs than relative MBF (*ρ* = −0.53 vs. *ρ* = −0.43). Previous studies generally support higher diagnostic accuracy by using relative MBF than absolute MBF in stress CCTP (27, 28). However, Kajander et al. reported a higher diagnostic accuracy by using absolute MBF than relative MBF with positron emission tomography (PET) (29). Another PET study by Stuijfzand et al. did not find significant improvement in detecting hemodynamically significant stenosis with relative MBF compared to absolute MBF alone (30). Possible explanations include subclinical atherosclerosis in reference territories, presence of diffuse ischemia in patients with multi-vessel disease, and the inherent noise and variations associated with ratio measurements of relative MBF. We found particularly poor correlation between relative MBF and CAD-RADS when we included patients having diabetes, suggesting that the presence of diffuse MVD can confound usage of relative MBF.

Using these methods, we identified a high prevalence of ischemia in patients without obstructive CAD, especially among diabetic patients (Figures 4,5). We found that in patients *without* obstructive stenosis, 15 out of 67 (22%) showed reduced MBF, suggesting an opportunity for deeper analysis. In Figure 5, for the 12 diabetic patients without obstructive stenosis (DM-CAD-RADS < 3), 10 had reduced MBF (indicating MVD) in one or more territories (83%) compared 5 patients (6%) in those without diabetes. MVD Studies show that MVD is prevalent in patients with symptoms of CAD (31, 32). In the ISCHEMIA trial, 13% of patients with moderate or severe ischemia confirmed by core lab did not show evidence of obstructive CAD on CCTA (31). A recent study also showed hyperemic MBF is significantly lower in territories with MVD, as confirmed by the index of microcirculatory resistance (33). Murthy et al. used PET to assess myocardial ischemia in 1,218 symptomatic patients without obstructive CAD and showed that 51% of men and 54% of women had MVD (32). From the MBF spatial distribution for patients with diabetes (Figure 5), 12/18 diabetic patients showed diffuse ischemia. Our finding confirmed that MVD is prevalent in diabetics, as identified by other quantitative methods (34, 35). The significantly lower MBF in patients with diabetes compared to normal patients was demonstrated in PET (34). Using intracoronary thermodilution technique, Gallinoro et al. showed higher microvascular resistance in patients with diabetes as compared to those without diabetes (35).

Our study has several limitations. It was conducted at a single site with a modest sample size (104 patients after exclusions), which may reduce generalizability. AHA segments were assigned assuming a right-dominant coronary distribution, which may not reflect individual variations in coronary anatomy and could introduce minor inaccuracies in per-territory MBF assessment. CAD-RADS was an anatomical grading system and used as the reference standard. Lesions with CAD-RADS ≥3 may not always be hemodynamically significant. To reduce this limitation, ROC analyses were restricted to unequivocal cases (CAD-RADS 0 and 4). However, invasive physiological validation remains the gold standard for identifying flow-limiting stenoses. Validation of CCTP against invasive testing will be essential in future studies. CT-derived FFR was not available and should be evaluated in future studies. The diabetic subgroup was limited, and detailed clinical data, such as diabetes duration, were unavailable, which may affect interpretation. Despite these limitations, combining MBF from CCTP with stenosis assessment from CCTA provides a comprehensive non-invasive evaluation, enabling better distinction between obstructive and microvascular disease.

## 5 Conclusion

Combining CCTA with a new automated quantitative CCTP approach enhances CAD interpretation, enabling differentiation between ischemia caused by obstructive lesions and microvascular disease.

## Data Availability

The datasets presented in this article are not readily available due to data sharing agreements and IRB protocols. Requests to access the datasets should be directed to the corresponding author.
