# Supplementary material for: Quantitative cardiac CT perfusion: physiologically-inspired model and identifying microvascular disease from discordant CTA CAD-RADS
Source: Front Cardiovasc Med. 2025 Nov 10;12:1621443. doi: 10.3389/fcvm.2025.1621443 (PMC12640952; doi:10.3389/fcvm.2025.1621443)
Supplement: Supplementary file 1 [file Datasheet1.docx]

**Supplemental material for “Quantitative cardiac CT perfusion blood flow using a physiologically-inspired model and identification of microvascular disease from discordant CTA CAD-RADS” Hao Wu, et al.**

**Supplemental methods**

**CT acquisition and reconstruction setting**

CCTA acquisition was performed with a dual source, 120 kVp, 320 mA per rotation, and a 0.28-second gantry rotation time. Prospective ECG triggering covered 70- 80% of the R-R interval. Images were reconstructed with a medium smooth kernel (B26), a slice thickness of 0.5 mm, and an increment of 0.3 mm.

In CCTP, scan parameters were dual source, 100-kVp, 320-mAs per rotation, and two alternating table positions were used in the prospective ECG-triggered mode. The table moved forward and backward between the two positions in “shuttle mode,” with 300 mm/sec table acceleration. As the detector width was 38 mm with a 10% image overlap at the two positions, the imaging coverage was 73 mm. Voxel size was ~0.4 mm in plane with 3 mm slice thickness. Images were acquired in every single heartbeat for a heart rate less than or equal to 63 beats per minute and every second heartbeat for a heart rate greater than 63 beats per minute.

**Quantitative CT perfusion pipeline**

The quantitative CT perfusion pipeline includes (1) aorta ROI detection, (2) scan-scan registration, (3) myocardium segmentation, (4) automatic beam hardening correction, (5) MBF computation on axial images, and (6) MBF polar map conversion. The whole pipeline was implemented using MATLAB 2022b on a computer with an Intel Core i7-8700 CPU, 3.20GHz, 64 GB RAM, and NVIDIA GTX 1080TI GPU. We trained convolutional neural networks for myocardium segmentation and landmarks (interventricular septum and center of left ventricle) detection in MBF polar map conversion. Two expert analysts manually annotated the CCTP images using AMIRA (Thermo Fisher Scientific Inc). Myocardium was segmented on the peak enhancement volume in axial images. The landmarks for polar map conversion were labeled on peak enhancement volume in the short-axis. Analyst1 labeled the entire dataset, including 104 patients, to train the two convolutional neural networks. Analyst2 labeled 15 patients to perform an inter-observer study and evaluate the performance of the pipeline.

1. **Aorta ROI detection.** We first performed an aorta ROI detection for the CCTP data to identify the peak enhancement time point of the aorta for registration of the CCTP data. The processing step of the aorta ROI is shown below.
   1. We defined the initial slice as the middle slice of the volume at the middle time point (Number of scans/2). A template-matching algorithm was applied to find the bounding box of the aorta. We obtained ten cropped images of the aorta as the templates from different patients, and all the templates were obtained from the middle slice at the middle time point. For each template, the algorithm then found the optimized location for the bounding box of the template by minimizing the normalized correlation between the template and the image inside the bounding box. The final location of the bounding box was the average of the output of those ten templates.
   2. We fitted a circular shape into the image with the rectangular mask based on the Hough transform to obtain the ROI of the aorta at the current time point. The center coordinate of the circular ROI was passed to the next time point.
   3. A bounding box with the same size as step (a) was generated for the image slice at the next time point. The center coordinate of the bounding box was received from (b). Then a circular shape ROI fits into the image as (b).
   4. Repeat steps (b) and (c) until go through all time points for the current slice.

The aorta's time curves were obtained based on the average intensity inside the ROI at different times. The time point of the peak enhancement of the aorta was obtained from the time curve of the aorta.

1. **Scan-scan registration.** We performed a two-step registration to reduce the motion in CCTP images. We first identified the time point of aorta peak enhancement and used the volume at the time point as the fixing volume. All volumes at other time points were considered moving volumes that need to be registered. We used a rigid-body registration for each volume to reduce breathing motion, followed by a non-rigid registration (1) to reduce heart motion. We used normalized mutual information for both steps to measure the similarity between volumes.
2. **Segmentation of myocardium and aorta.** We used a modified 2D U-Net architecture to perform myocardium and aorta segmentation. The network contains an encoding path with 4 down-sampling layers with stride convolutions and a decoding path with 4 up-sampling layers with transpose deconvolutions. The encoder and decoder are connected by skip connection. The network was modified to operate on 256x256x3 voxel sub-volume input and output the myocardium and aorta segmentation predictions for the middle slice. The three consecutive slices input helps the network to consider the textural information from adjacent axial slices. To emphasize the iodine and myocardium structure in images, we first cropped the intensity range of images to [0, 300-HU], then linearly rescaled the intensity range to [0, 1]. The network was trained with Adam optimizer, with an initial learning rate of 0.001. The loss function was defined as the negative sum of the DICE coefficient overall class. After obtaining the segmentation prediction, morphologic operations were performed to remove small structures in each class. Each class's largest connected component in 3D was considered the final output. The detail of the training and testing process can be found in the supplement material.

We manually segmented the myocardium and aorta from volumes at the time point of the aorta at the peak enhancement. To enrich our dataset, we registered the two volumes before and after the peak enhancement to the peak enhancement volume and used the same masks. We performed 10-fold cross-validation for 104 patients (312 volumes). In each of the 10-fold, patients were separated into training, held-out validation, and held-out testing by 80%, 10%, and 10%. For each fold, patients in the validation set were used to determine the stopping criterion of training. For patients in the hold-out test set of each fold, the myocardium and aorta were segmented by an independent model. To quantitatively evaluate the segmentation, we measured the Dice score between the predicted and the manual segmentation for the 10% held-out patients in each fold.

1. **Automatic beam hardening correction (ABHC).** We performed image-based automatic beam hardening (BH) correction based on our previous work (2). Briefly, we used input CT images and iteratively optimized parameters in a polynomial BH correction until a BH-sensitive cost function was minimized on output images. The input image was segmented into a soft tissue image and a highly attenuating material image containing bones and regions of high iodine concentrations. We forward projected HAM, corrected projection values according to a polynomial correction, and reconstructed a correction image to obtain the current iteration's BH corrected image. The cost function was sensitive to BH streak artifacts and cupping.

To apply ABHC to dynamic perfusion images, we first preprocessed the images. We register the cardiac volumes obtained over time using the scan-scan registration method described above for ECG-gated image acquisitions. The segmentations of the myocardium and aorta were used for calculating the cost function. We identified the peak enhancement image, estimated parameters at this image and the two adjoining images in time, estimated parameters for each image and averaged them. Then the averaged parameters were applied to correct all other images.

1. **MBF computation on axial images.** A previously developed SLICR algorithm that combines a modified simple linear iterative clustering algorithm (SLIC) with a robust physiologic model (RPM) was used to estimate myocardial blood flow (MBF) maps from dynamic perfusion images (3,4). The steps of SLICR are: i) create super-voxels from voxels close in space with similar temporal characteristics, ii) generate, for each super-voxel, a TAC using a robust estimator, a-trimmed-mean, at each time point, and iii) estimate MBF for each super-voxel TAC using our previously reported RPM method.

SLICR was demonstrated to be computationally efficient meanwhile enable accurate, precise MBF measurement over a range of clinically relevant imaging conditions and hemodynamic states (4). RPM is a reduced form of the Johnson-Wilson tissue homogeneity model, which uses prior knowledge of contrast agent extraction and tissue physiology to constrain the extraction fraction and intravascular transit time parameters. Three free parameters are in RPM; the time delay, MBF, and decay constant. Parameter estimation was done using a gradient-based optimization algorithm with a semi-analytic implementation of convolution, a method that has been reported to provide rapid and reliable convergence to globally optimal parameters (3).

1. **MBF polar map conversion.** We used the free software Synedra viewer (Synedra information technologies gmbh, Innsbruck, Austria) to performed short-axis reformatting for the axial images. The short-axis reformatting was applied to the volume at the time point of peak enhancement. Then, the geometric transformation for the short-axis slice was applied to both MBF data and the myocardium mask with a 2mm slice thickness. We used bi-cubic interpolation and nearest neighbor interpolation for the MBF data and the myocardium mask, respectively.

We performed a landmarks detection method to identify (i) the center of the LV, (ii) the upper, and (iii) the bottom septum to generate MBF polar map that can align with the AHA segments model. We established septum detection on short-axis images using a modified 2D U-NET architecture. For each short-axis slice, the three landmarks were manually labeled separately. For each volume, ~30 slices were labeled. For the mask of each landmark, we applied a 2D Gaussian filter with sigma = 5 to generate a blurred version of the landmarks depicted in Fig S2. We treated the localization of these landmarks as a regression problem. We used a similar 2D U-Net architecture to the myocardium segmentation task. The network was modified to operate on 256x256x3 voxel sub-volume. The output was a 256x256x3 prediction of the 3 Gaussian blurred landmarks for the middle slice. The loss function was the mean square error between the prediction and the blurred landmarks. For each landmark prediction, we extracted the pixels in the prediction image above a threshold = 0.7, and the largest component's mass center was considered the predicted landmark.

We trained and tested the model using the same strategy used in myocardium segmentation. We performed a 10-fold cross-validation of the whole dataset. The 104 patients were separated into training, validation, and testing by 80%, 10%, and 10%, respectively. For patients in the hold-out test set in each of the 10-fold, the landmark was predicted by an independent model.

Once the landmarks in short-axis data were obtained, we performed a preprocessing pipeline to generate an MBF polar map from short-axis MBF images. The preprocessing steps are described below:

1. We rotated the short-axis image around the center to align the upper septum to 11 o’clock. If the upper septum was not presented on the short-axis images due to the coverage of the CCTP scan, we rotated the short-axis image around the center to align the bottom septum to 7 o’clock. Users can also check the landmarks and correct them manually.
2. We generated an r-θ image for each short-axis slice by using the center of LV as the origin. A voxel in the volume $V(x_{v},y_{v},z)$ was converted to $V(r,\theta,z)$ by interpolation of each slice, where $x_{v}=r\times cos(\theta)$, $y_{v}=r\times sin(\theta)$, and $z$ is the slice number.

For a pixel on the polar map $P$, we can represent it as$P(z,\theta)$ under the polar coordinate. The relationship between a pixel on the polar map $P$ and a volume $V$ is described mathematically and depicted in Fig S1:

(1)

where $f$ is the function that computed the α-trimmed mean (α = 0.1) of MBF in voxels from the endocardium to the epicardium along the *θ*. To fill pixel values in a gird to generate the polar map, a pixel on the polar map in Cartesian coordinate is:

$P(x_{p},y_{p})=P(z,\theta)=f_{r=endo}^{epi}(V(r,\theta,z),a)$ (2)

where $z=\sqrt{{x_{p}}^{2}+{y_{p}}^{2}}$, and $\theta={tan}^{-1} (\frac{y_{p}}{x_{p}})$. Since we convert the short-axis volume from $V(x_{v},y_{v},z)$ to $V(r,\theta,z)$ previously, the pixel on the polar map $P(x_{p},y_{p})$ can be directly obtained by interpolating the $V(r,\theta,z)$.

The MBF on the polar map was then applied to the modified 16 segments of the American Heart Association (AHA) model by excluding the apex (20). To map the MBF on the polar map to the AHA model, we used the alpha trimmed mean (α= 0.1) of MBFs of pixels on the polar map to represent the MBF of the corresponding segment. We excluded the segment from the AHA model if the corresponding pixel number on the polar map is less than 30% of the segment area. The representative MBF of each territory (LAD, RCA, and LCX) was obtained by averaging the two connected segments with minimum MBF in the territory.


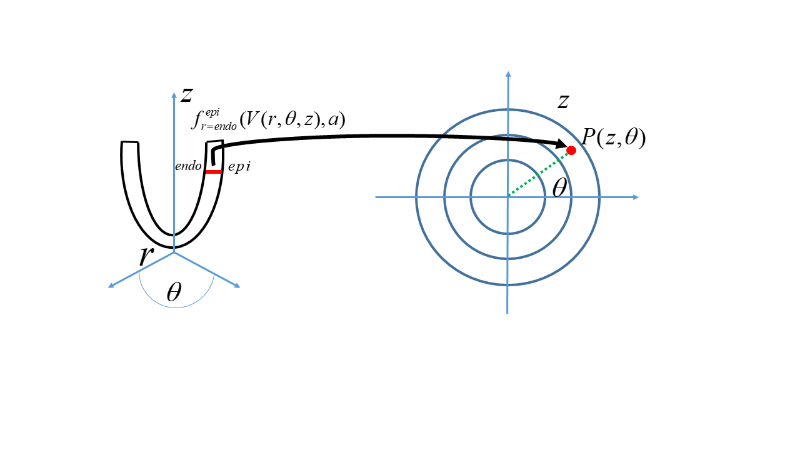


Figure S1. Mapping from short-axis volume to polar map. For a pixel (red point) in polar map, the value of the pixel equations to the a-trimmed mean of a line of pixels (red line) from endocardium to epicardium in the volume.

**Supplemental results**

**Myocardium and aorta segmentation.** We quantitively evaluated each intermediate and final output of our software. For the automated segmentation module for myocardium and aorta, the average Dice score was 0.90±0.04, and 0.92±0.02, respectively. Mis-segmentation of the myocardium mainly occurs around the apex (Fig S2), where partial volume artifacts and possibly anatomy give an apparently thin myocardium. In our modified AHA-16 model, we excluded the apex for MBF analysis.


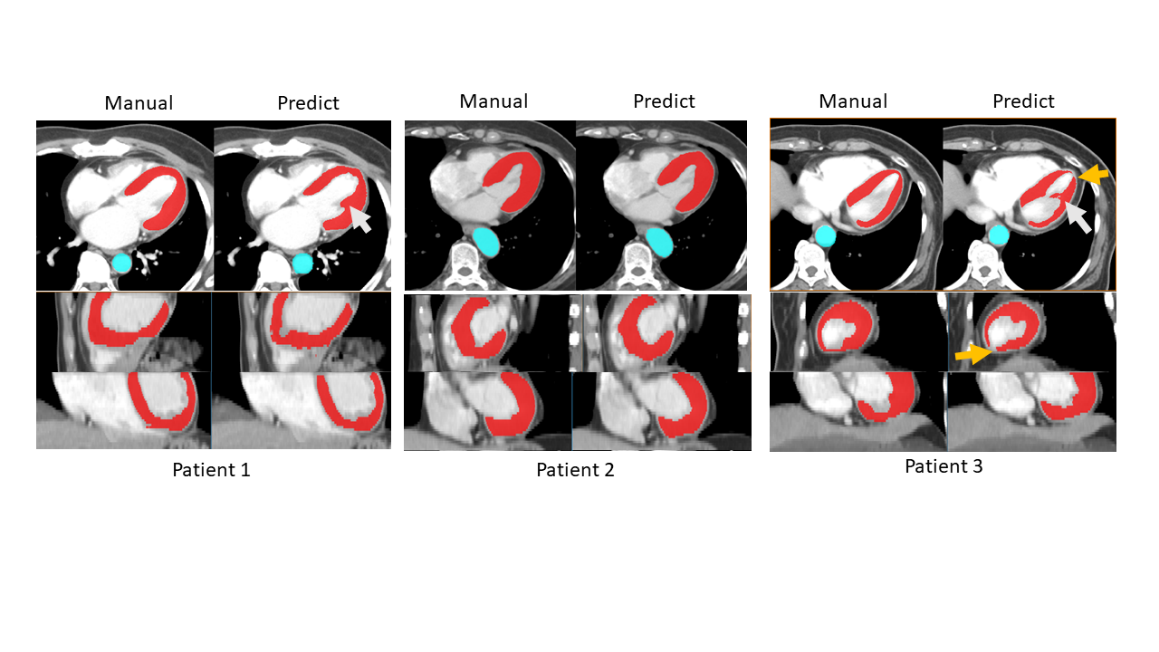


Figure S2. Segmentations on axial (top), sagittal (middle), and coronal (bottom) views of three patients. The DICE scores of the myocardium/aorta are 0.91/0.94, 0.92/0.95, and 0.86/0.91 for patients 1, 2, and 3, respectively. In patient 3, missegmentation can be found around the apex due to the thin myocardium (yellow arrows). The papillary muscle in the left ventricle was also segmented in patient 1 and patient 3 (white arrows).

**Ventricular septum and center localization.** We quantitatively evaluated the prediction of ventricular landmarks by measuring the Euclidean distance between predictions and manual landmarks of each slice for the held-out test patients in each fold. Euclidian distances were 2.3±1.9mm, 2.9±4.1mm, and 3.8±2.7mm for the center of LV, upper septum, and bottom septum, respectively (Fig S3).


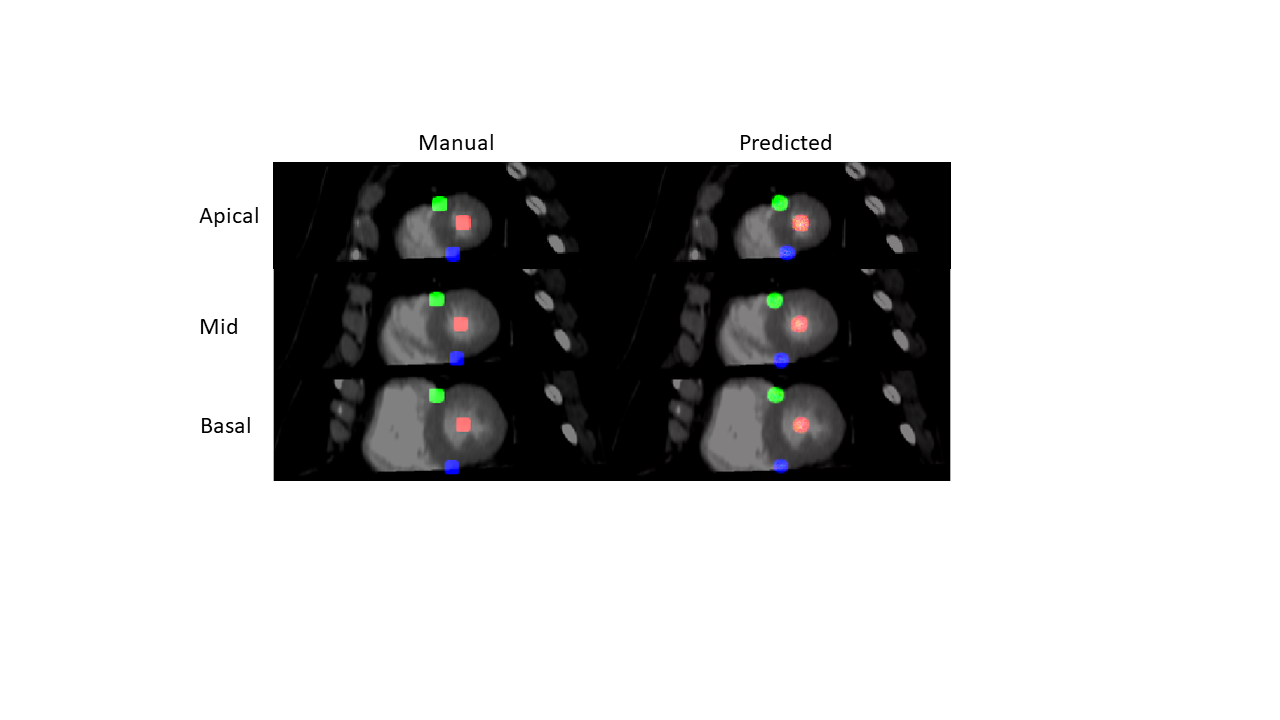


Figure S3. Landmarks localization on apical (top), mid (middle), and basal (bottom) in short-axis views of a patient. The manual labels were blurred by a Gaussian filter for visualization and for the training process. The second column shows the output of the regression model. The Euclidean distance between the manually labeled and predicted landmarks are 1.5±0.5mm, 1.8±1.9mm, and 5.6±3mm for the center of LV, upper, and bottom septum. (Red: center of LV, green: upper septum, blue: bottom septum).

**Performance comparison between automated and manual pipelines**. We evaluated our proposed pipeline's performance by comparing the final AHA-16 segments to those generated by a manual pipeline using the 15 patients annotated by both expert analysts. In the manual pipeline, analysts manually performed (i) myocardium segmentation, (ii) short-axis reformatting, and (iii) landmarks detection. We analyzed the variation between analysts (inter-reader variability) (Fig. S4) and compared the variation between automatic and manual pipelines (Fig. S5). The scatter and Bland-Altman plots between the two analysts are shown in Fig. S4. There was reasonable agreement (slope (R) =0.99, p<0.001) between the two analysts; however, the standard-deviation/bias from the Bland-Altman plot (6.7/2.4 mL/min-100g) indicated variability. The largest outlier showed a difference of -18 mL/min-100g out of 178 mL/min-100g, a 10% difference. Inspection of the myocardium segmentation and landmarks, expert analysts disagreed on the placement of the myocardium boundary due to the heart's motion. Fig. S5 shows scatter plots comparing the segmentation from the same two analysts against the automatic pipeline for the same 15 patients. There was good agreement with both analyst1 and analyst2 (R=0.98, p<0.001 and R=0.98, p<0.001, respectively)


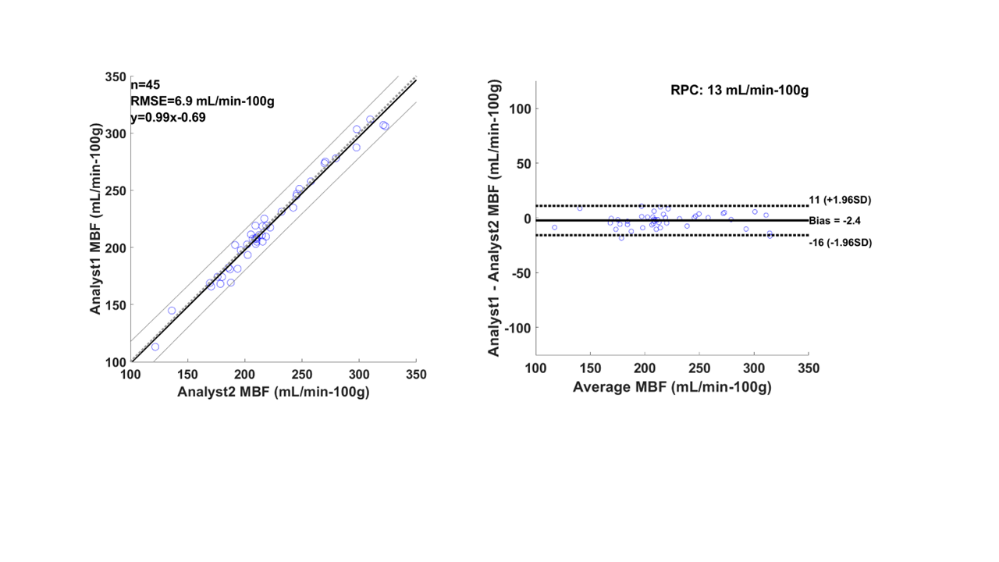


Figure S4: Comparison of territory-based MBFs by using manual pipeline from analysts 1 and 2. Good agreement is observed between analysts 1 and 2 in both the scatter plot and Bland-Altman plot. The slope is 0.99 between the two analysts. Bias is small (2.4 mL/min-100g), only 1-2% of the measured MBFs.

**
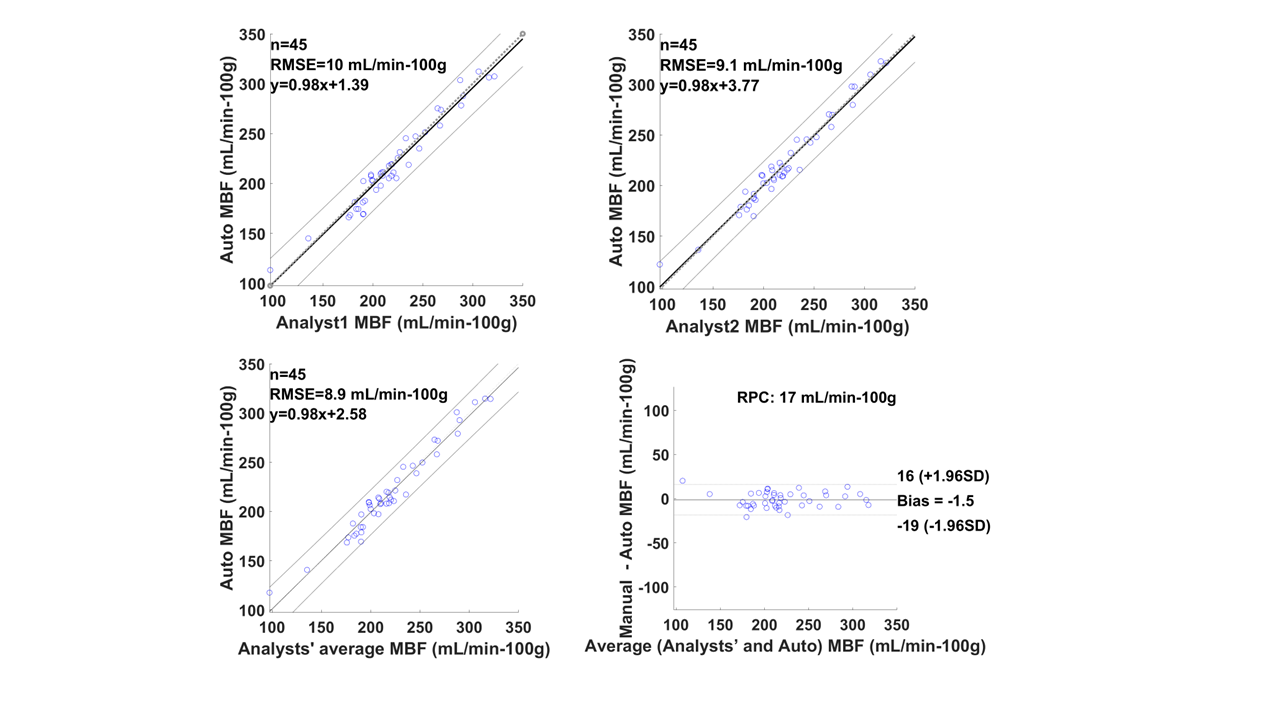
**

Figure S5. Comparison of territory-based MBF by automatic pipeline and manual pipeline from the two analysts. A similar correlation is found with Analyst1 (A) and with Analyst2 (B), the slopes are 0.98 in both. C and D compare automatic pipeline to MBF averaged for manual pipelines from Analyst1 and Analyst2. The scatter plot of automatic pipeline MBF against the average of Analyst1 and Analyst 2 (C) is visually comparable to that between Analyst1 and Analyst2 in Fig. S4, indicating that the automatic pipeline performs well compared to analysts. Bland-Altman plot (D) compares favorably to that for Analyst1 versus Analyst2 in Fig. S4. The average - automatic shows a 38% reduction in bias compared to Analyst2-Analyst1. The spread with average- automatic is only a little larger (30%) than that for Analyst2-Analyst1.

**MBF cutoff determination.** We determined an MBF threshold for abnormal flow by analyzing non-diabetic patients with unequivocal absence or presence of stenosis (CAD-RADS 0 or 4. Patients with diabetes were excluded to reduce any confounding by MVD. Randomly selecting 30 territories per score, we performed a bootstrapping-corrected ROC analysis with 1000 bootstrapped samples, using CAD-RADS=4 as the true positive for abnormal flow (Fig. S6-right). The optimal MBF threshold, determined by the mean Youden index, was 200 mL/min-100g.


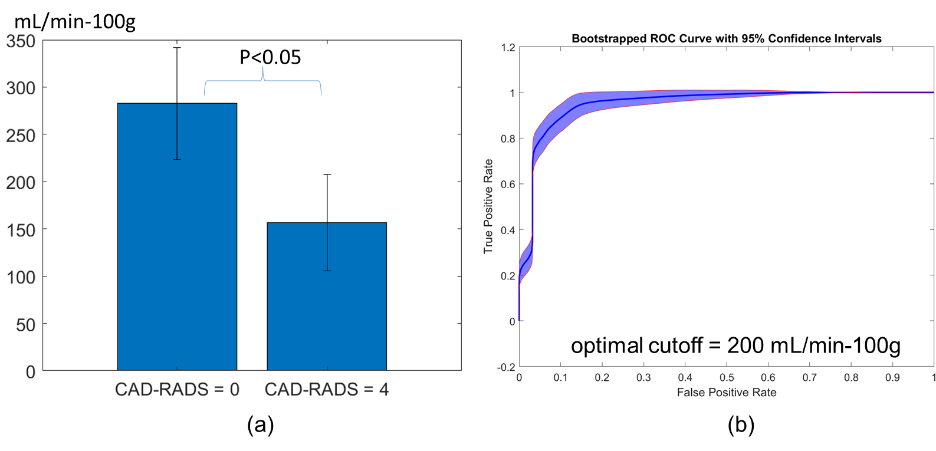


Figure S6. Obtaining an “optimal” MBF cutoff based on an analysis of unequivocal CAD-RADS cases. In (a), territory MBFs are much lower with CAD-RADS = 4 than with CAD-RADS = 0 (163 ± 37 V.S. 284 ± 58 mL/min-100g, respectively, p<0.05). In (b), assuming CAD-RADS = 4 to be (+), a ROC curve with AUC=0.95 (95% confidence interval: 0.90 – 1.0) was created by sweeping a cutoff across MBF values. The “optimal” threshold (200 mL/min-100g) for abnormal MBF was obtained by the Youden index; the 95% confidence interval was 179.2 - 220.8 mL/min-100g. Diabetic patients were excluded, to reduce potential confounding by microvascular disease.


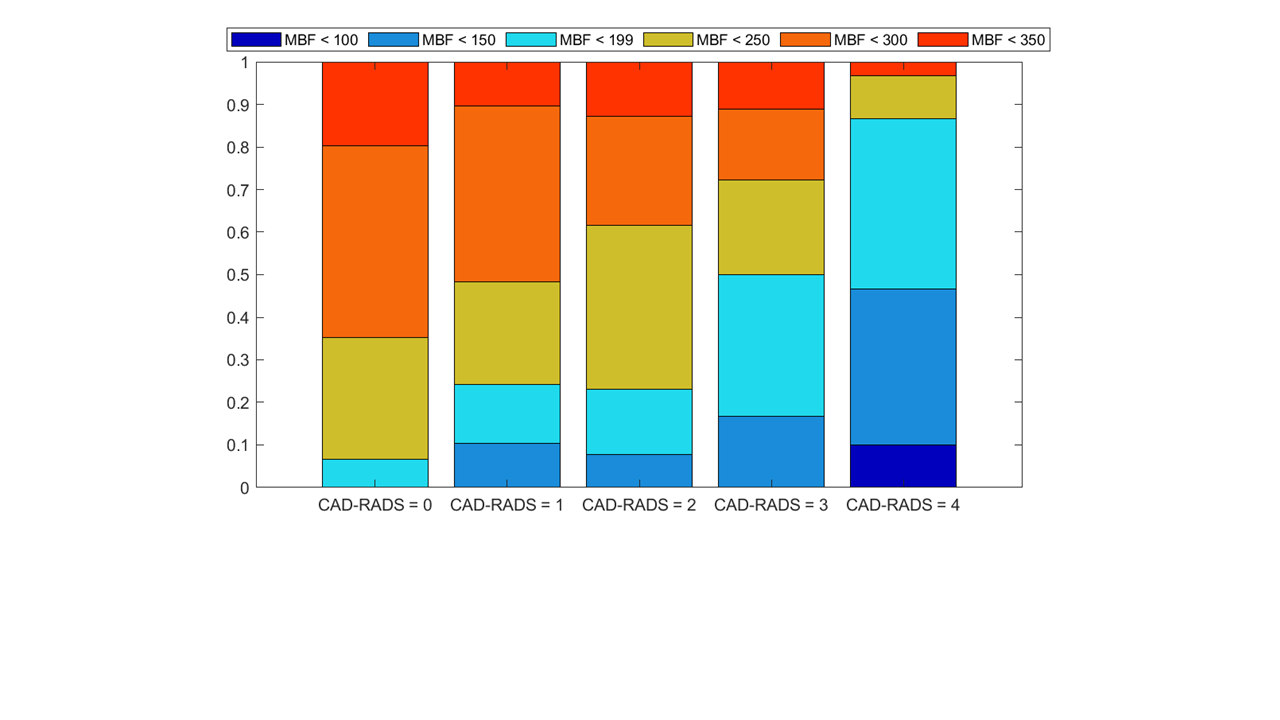


Figure S7. Percentage of individual territory MBF categorizations as a function of CAD-RAD scores for obstructive disease. Diabetic patients are excluded to reduce the confounding of microvascular disease. For territories with CAD-RADS = 0, 1, and 2, 96%, 79%, and 80% of territories show normal (≥200 mL/ min-100g) or greater MBF. 65%, 52%, and 38%, respectively, have MBF >250 mL/ min-100g. For these instances, no territories have MBF < 100 mL/ min-100g. For territories with CAD-RADS = 3, and 4, 49%, and 13%, respectively, have MBF ≥ 200 mL/min-100g. Percentages of high MBF values (>300 mL/ min-100g) drop precipitously as one proceeds from left to right. Altogether, the percentages falling in different groups are consistent with the extent of vascular obstruction.

**Reference**

1. Modat M., Ridgway GR., Taylor ZA., et al. Fast free-form deformation using graphics processing units. Computer Methods and Programs in Biomedicine 2010;98(3):278–84. Doi: 10.1016/j.cmpb.2009.09.002.

2. Levi J., Wu H., Eck BL., et al. Comparison of automated beam hardening correction (ABHC) algorithms for myocardial perfusion imaging using computed tomography. Medical Physics 2021;48(1):287–99. Doi: https://doi.org/10.1002/mp.14599.

3. Eck BL., Muzic RF., Levi J., et al. The role of acquisition and quantification methods in myocardial blood flow estimability for myocardial perfusion imaging CT. Phys Med Biol 2018;63(18):185011. Doi: 10.1088/1361-6560/aadab6.

4. Wu H., Eck BL., Levi J., et al. SLICR super-voxel algorithm for fast, robust quantification of myocardial blood flow by dynamic computed tomography myocardial perfusion imaging. JMI 2019;6(4):046001. Doi: 10.1117/1.JMI.6.4.046001.
